# *Trichoderma asperellum* and *T. asperelloides*: Comparative Genomic Study for Genes Implicated in Biocontrol and Biofertilizer Activities

**DOI:** 10.3390/jof12060418

**Published:** 2026-06-09

**Authors:** Adnan Ismaiel, Jackson Maul, Patricia Millner

**Affiliations:** Sustainable Agricultural Systems Laboratory, The United States Department of Agriculture-Agricultural Research Service, Beltsville, MD 20705, USA; jackson.maul@usda.gov

**Keywords:** chitinases, cellulases, proteases, NRPS, PKS, ITS, mycoparasitism, plant growth promotion

## Abstract

*Trichoderma asperellum* and *T. asperelloides* are two cryptic species that have potential for use as biocontrol and biofertilizer (B&B) agents. Comparison of the reference genomes of the two species revealed that each species had seven chromosomes, but *Trichoderma asperellum* has about 1000 more genes than *T. asperelloides*. The number of genes coding for chitinases, cellulases, xylanases, secreted proteases, and genes involved in soil and plant health was slightly greater in *T. asperellum* than in *T. asperelloides*. Moreover, *T. asperellum* had five more genes than *T. asperelloides* involved in the synthesis of secondary metabolites like peptaibols and siderophores. The B&B genes were distributed on all the chromosomes. No duplicate genes were found for any of the enzymes searched. The investigation also revealed that *T. asperellum* had 15 copies of the internal transcribed spacer (ITS) region of ribosomal DNA compared to only seven copies in *T. asperelloides*. Further transcriptomic, proteomic, and efficacy studies are needed to determine the impact of the missing genes in *T. asperelloides* on its B&B activities compared to those of *T. asperellum.* The search for B&B genes in *T. asperelloides* was hindered by the lack of annotation for the genome. Thus, comparison only involves B&B genes searched in *T. asperellum* and whether homologs to the genes were available or missing in *T. asperelloides.* A comparison between additional strains of the two species is essential to show whether the data in this study apply to all intraspecies strains of the two species.

## 1. Introduction

Species in the genus *Trichoderma* are cosmopolitan in soil and are known for various biochemical and biological activities significant in agriculture [1], industry [2], and medical fields [3]. In this study, the focus is on the use of *Trichoderma* in agriculture as biocontrol against a wide range of plant fungal pathogens and as a promoter of plant growth and health. Biocontrol has gained increased attention due to the adverse effects of synthetic pesticides on the environment and health, such as resistance to chemical pesticides, the modification or eradication of natural microbiota, the distortion of the natural habitats of plants, soil contamination, and the bioaccumulation of hazardous chemicals [4].

Since the early 1930s, the importance of *Trichoderma* for biological control against fungal diseases has been known when *Trichoderma lignorum* (later recognized as *T. atroviride*) was revealed as a parasite of other fungi [5]. About four decades later, *Trichoderma* was also reported to enhance plant growth and yield. Lindsey and Baker [6] reported increased weight and height of dwarf tomato plants after treatment with *T. viride*. Similarly, Chang et al. [7] reported enhanced germination, rapid flowering, and increased height and weight of three different plants upon treatment of their soil with *T. harzianum*. In the 1990s, *Trichoderma*-based products, such as bio-fungicide and biofertilizer, were commercialized in both developed and developing countries with reasonable success [8,9].

Selecting effective species/strains from among the more than 550 species of *Trichoderma* (based on data in www.SpeciesFungorum.org, accessed on 11 May 2026) requires significant experimental effort to screen the performance of numerous isolates. Genomic comparison of key activities associated with biocontrol can aid selection of superior strains and thereby accelerate the introduction of more *Trichoderma*-based products in agriculture. Ismaiel et al. [10] analyzed data from 23 survey studies of *Trichoderma* species isolated from soil spanning different global regions, showing that the well-recognized species for biocontrol use, such as *T. atroviride* and *T. virens*, are also the dominant and commonly isolated species from soil. Such findings suggest that successful competition for space and nutrients in situ may be an important mechanism in the biocontrol activity of these species. The two cryptic species, *T. asperellum* and *T. asperelloides,* described by Samuels et al. [11,12], were also commonly isolated from soil [10]. In a survey study of *Trichoderma* from South and Central America, 60 of 183 isolates were *T. asperellum/T. asperelloides*, which was more than all other species isolated in the study [13]. In another large-scale study on the presence of *Trichoderma* species in soils from a different region, China, *T. asperellum* strains were the second most frequently isolated (425) compared to *T. harzianum* (429) [14]. The two species, *T. asperellum and T. asperelloides*, were designated as sister cryptic species because there were no phenotypic differences between them, such as growth on two media, shape of conidia, and other fungal structures. However, cryptic species may differ in their activities, biogeographic distribution, and host [15]. Both *T. asperellum* and *T. asperelloides* species are cosmopolitan. However, based on data for deposited sequences in GenBank, the most prevalent countries of origin for the isolates of these two species were tropical regions in Malaysia, Brazil, China, and India [10]. Samuels and Hebbar [16] reported that *T. asperellum* grew better at 35 °C than one of the most widely used species in biocontrol, *T. atroviride*. The maximum radial growth at 35 °C on potato dextrose agar and spezieller nährstoffarmer agar (synthetic nutrient-deficient agar) in Petri dishes was 43–45 mm for *T. asperellum* after 72 h of incubation versus 7–8 mm for *T. atroviride* [16]. The higher growth rate in the media could provide an explanation for the high frequency of isolation of *T. asperellum*/*T. asperelloides* in warm climates relative to *T. atroviride*.

Both *T. asperellum* and *T. asperelloides* exhibit antifungal activities, plant growth promotion, and stress resistance in various crops, as summarized in Table 1. Additional biocontrol study data for *T. asperellum* are available [17]. Consequently, there are three commercially available products with *T. asperellum* as the active ingredient in European markets with trade names: Remedier, Tenet, and Tusal [18].

The mechanisms used by the *Trichoderma* species for biocontrol include antibiosis, mycoparasitism, induced systemic resistance, and competition for space and nutrients [40,41,42,43]. Regardless of the mechanism, *Trichoderma* species apply lytic cell wall-degrading enzymes (CWDEs), e.g., chitinases, cellulases, proteases, and secondary metabolites as active measures against their prey. *Trichoderma* spp. are well-known for the production of various secondary metabolites. Non-ribosomal peptides and polyketides represent a major portion of these products [44]. Peptaibols are a large family of linear, amphipathic polypeptides consisting of 5–20 amino acid residues synthesized from the fungal non-ribosomal peptide synthetases (NRPS) pathway [45]. Additionally, Hou et al. [45] provided a list of peptaibols from different species of *Trichoderma* with antimicrobial activities against various fungi and bacteria. Peptaibols have also been implicated in plant defense-stimulating activities [46]. Specifically, *T. asperellum* produces at least two peptaibols identified as acid trichotoxin and neutral trichotoxin, which have an inhibitory effect on the growth of *Bacillus stearothermophilus* [47]. *Trichoderma* spp. are also known to a lesser extent for the production of polyketides (PKs), which are synthesized by the action of multifunctional enzymes polyketide synthases (PKSs). Direct impact of PKS in biocontrol was evident as deletion of the *Pks4* gene in *T. reesei* reduced its antifungal ability on three fungal pathogens [44]. In a recent report, genes coding for chitinases, glucanases (cellulases), β-glucosidases, and genes involved in antibiosis, e.g., NRPS, PKS, in *T. harezianum* T4 were all found to be significantly upregulated during and after contact with the phytopathogen *Rhizoctonia solani* compared to the period before contact, revealing their direct role in mycoparasitism [48].

Isolation, identification, and manipulation of lytic enzymes and secondary metabolites involved in biocontrol via mutants is one way to assess the biocontrol potential of *Trichoderma* strains [49,50,51,52]. Another alternative method is to search the whole genome for the genes coding for CWDEs and for the synthesis of secondary metabolites involved in biocontrol, with the premise that more of these genes enhance possible biocontrol effects of these species or strains. In this investigation, a comparative study of the genome assembly of *T. asperellum* and *T. asperelloides* was conducted with a focus on genes coding for enzymes involved in B&B, specifically, chitinases, cellulases, proteases, xylanases, enzymes for the synthesis of non-ribosomal peptides and polyketides, and genes coding for enzymes involved in soil improvement and plant growth promotion.

Additionally, it is well-known that in fungi, including *Trichoderma*, the ITS region of the rDNA exists in tandem with all the genes for rRNA: 18S, 5.8S, and 28S on the genome. Thus, the number of ITS copies in the genome is proportional to the rRNA genes involved in the synthesis of ribosome sites needed for protein synthesis. We determined the copy number of the ITS region in the genomes of *T. asperellum* and *T. asperelloides*, their locations, and examined the possibility of a positive correlation between the copy number of the ITS region and their biocontrol activities.

## 2. Materials and Methods

### 2.1. Comparison of the Whole Genomes

**Figure 1:**
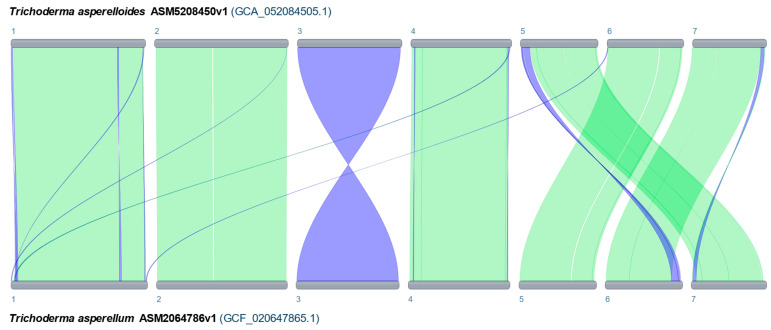
Comparison of *T. asperelloides* and *T. asperellum* chromosomes DNA sequences (diagram generated by Comparative Genome Viewer of NCBI). Green and purple refer to homology in the same and opposite directions, respectively. The white lines represent unaligned regions.

The genomes of *Trichoderma asperellum* and *T. asperelloides* available in GenBank were compared using the Comparative Genome Viewer (CGV) tool of the National Center for Biotechnology Information (NCBI) available at https://www.ncbi.nlm.nih.gov/cgv (23 March 2026) (Figure S1) with the default parameters. The two whole-genome assemblies available in GenBank used in the comparison were: *Trichoderma asperellum* ASM2064786v1 for strain FT101 isolated from soil, Taiwan, in 2004 [53], and *Trichoderma asperelloides* ASM5208450v1 strain X01119, isolated from soil in the Yancheng region, China, in 2022. Both assemblies are reference genomes for the two species that have been assembled to the chromosome level. A diagram of the comparison was created (see Figure 1), revealing the homology and differences between the chromosomes of the two species. Then the seven homologous chromosome pairs were aligned, and % identities between them were determined using the Basic Local Alignment Search tool (BLAST) of the NCBI available at https://blast.ncbi.nlm.nih.gov/Blast.cgi (5 January 2026). Other general comparisons, like the size of the genomes and chromosomes, GC content, and total number of genes, were obtained when assemblies were viewed using the NCBI’s datasets tool available at https://www.ncbi.nlm.nih.gov/datasets/genome (20 March 2026).

### 2.2. Chitinases

The genome assembly for *T. asperellum* referred to above was opened in the NCBI tool “Genome Data Viewer (GDV)” available at https://www.ncbi.nlm.nih.gov/gdv (5 January 2026) (Figure S2). We entered the term “Chitinase” in the search bar. The search returned 22 genes along with information about the genes, e.g., code name, the chromosome location, the nucleotide range on the chromosome, and the number of amino acids. From the GenBank record of each gene, the glycosyl hydrolase (GH) family of genes was recorded and was cross-checked at the “Glycoside Hydrolase family classification” website available at https://www.cazy.org/Glycoside-Hydrolases.html (5 January 2026). Selecting (by double clicking on any gene code name) displayed the gene exons in thick green lines with arrows and introns in thin black lines. Selecting the gene (thick green line) displayed other options, allowing the choice of BLAST nucleotide at https://blast.ncbi.nlm.nih.gov/Blast.cgi (5 January 2026) (Figure S3). Within BLAST (under default parameters), we selected the option “Align two or more sequences”. Chromosome accession number for *T. asperellum* with the nucleotide range for the gene was used as a query against the whole sequence of corresponding homologous chromosomes in *T. asperelloides* (Figure S3). Once BLAST finished, we selected the results and, if present, we recorded the location of the gene on the chromosome, the nucleotide range, percent identity, and direction, with Plus/Plus used to designate that the genes in both species are in the same direction, whereas Plus/Minus was used to indicate that the two genes in the two species were in different directions. In all cases, the BLAST showed that there was one homolog for each gene with an E value of zero, a cover of 100%, and that the identities were above 95%. To determine if the different chitinase genes were homologous, the DNA sequences of all chitinase genes in *T. asperellum* were downloaded in a FASTA format and aligned using the Clustal Omega multiple sequence alignment tool available at https://www.ebi.ac.uk/jdispatcher/msa/clustalo (5 January 2026).

### 2.3. Cellulases, Xylanases, and Secreted Proteases

The genome of *T. asperellum* referenced above (opened in GDV) was searched for cellulase, 1, 4-beta-D-glucanase, endoglucanase, and exoglucanase (all synonyms of cellulases). We selected all matches that represented glycosyl hydrolases (GH) from different families of cellulases based on the Glycoside Hydrolases family classification available at https://www.cazy.org/Glycoside-Hydrolases.html (5 January 2026). We BLAST searched each gene sequence as a query against corresponding homologous chromosomes in *T. asperelloides* (accession numbers in Table 2) as the subject to determine the availability of the *T. asperellum* cellulase genes in *T. asperelloides*. The BLAST results were recorded as in the above section. To determine if the different cellulase genes in *T. asperellum* were homologous, the gene sequences of all the cellulases were downloaded in FASTA format and aligned using the Clustal Omega multiple sequence alignment (MSA) tool available as indicated in the above section.

The search for xylanases and secreted proteases in both *Trichoderma* species genomes was performed as described above for cellulases.

### 2.4. NRPS, PkS, and PkS-NRPS

Genes coding for nonribosomal protein synthetase (NRPS), polyketide synthase (PKS), and fused PKS-NRPS were searched in the *T. asperellum* genome using the abbreviations NRPS, PKS, and PKS-NRPS in the search, and then homologs to the genes in *T. asperelloides* were obtained following the steps described in the search for chitinases. We searched the GenBank record for every gene found in *T. asperellum* to determine if the record shows the words NRPS, PKS, and PKS-NRPS in it. We also looked at the GenBank record for each gene to see if the gene codes for a domain involved in the synthesis of siderophores. DNA sequences of different genes for NRPS, PKS, and PKS-NRPS in *T. asperellum* were downloaded and aligned as previously described (Section 2.3) to search for homology within the different genes or determine if any gene existed as duplicates.

Similarly, we compared the two species for genes coding for enzymes involved in soil improvement and plant growth. The genes compared were NADPH oxidase, involved in root growth; laccases, supporting lignin metabolism and assisting in biodegradation, including the detoxification of phenolic pollutants and dyes; urease, converting urea into nitrogen forms usable by plants; and acyl esterases, participating in the degradation of dead plant biomass in soil. In this study, all the genes of biocontrol and biofertilizer were searched in the *T. asperellum* genome, and then all the sequences of genes were BLAST searched separately against the sequences of *T. asperelloides* chromosomes to determine their presence or absence in the *T. asperelloides* genome.

### 2.5. Internal Transcribed Spacer (ITS) Region of Ribosomal DNA

The number of internal transcribed spacer (ITS) regions of rDNA was determined in the *T. asperellum* genome using the following steps: (1) In GenBank, we searched for “*Trichoderma asperellum* ITS.” The first match was the ITS sequence for the type strain of *T. asperellum,* strain CBS 433.97 (accession number: NR_130668.1). (2) The genome assembly indicated in Section 2.1 for *T. asperellum* (ASM2064786v1) was searched in GenBank and opened (https://www.ncbi.nlm.nih.gov/datasets/genome/GCF_020647865.1/, 5 January 2026). (3) We selected the “BLAST the reference genome” option (Figure S4), which takes the assembly sequence to BLAST at the NCBI website (https://blast.ncbi.nlm.nih.gov/Blast.cgi, 5 January 2026) (Figure S5) as the database for the BLAST (Subject). In the space for a query sequence, we entered NR_130668.1, the accession number for the ITS region for the *T. asperellum*-type strain (CBS 443.97) determined in step 1. Then BLAST was selected. (4) Once BLAST processing was executed, we selected the results line and observed all the sequences of all the regions of the assembly sequence aligned with the ITS query sequence. (5) The sequences of all the copies were downloaded using the option “download in aligned FASTA format” and viewed with Mesquite, a modular system for evolutionary analysis, version 2.75 software. (6) Steps 1–5 were repeated to determine the ITS copies of *T. asperelloides.* (7) The sequences of all the ITS regions for both species were combined using Mesquite, and a phylogenetic tree was obtained by MEGA version 11.0.8 [54].

We emphasize here that for all the genes that were compared in this study (Section 2.2, Section 2.3, Section 2.4 and Section 2.5), we searched the genome of *T. asperellum* for those genes and then looked for their homologs in *T. asperelloides* using a BLAST search. Thus, the tables show only the genes present in *T. asperellum* and whether their homologs were present or missing in *T. asperelloides*. However, searching in the opposite direction (in *T. asperelloides)* was not possible due to a lack of annotation of the genome, and thus, the genes present in *T. asperelloides* but present or missing in *T. asperellum* were not studied.

## 3. Results

### 3.1. Comparison of T. asperellum and T. asperelloides Genomes

The whole genomes of the two species, *T. asperellum* and *T. asperelloides,* had sizes of 37.3 and 36.9 Mb, respectively (Table 2). Each genome consisted of seven chromosomes. The sizes of homologous chromosomes in the two genomes were comparable (Table 2). The total number of genes in the genomes of *T. asperellum* and *T. asperelloides* was 12,041 and 11,096, respectively. The identity between any two homologous chromosomes was about 95%. Similarly, the GC% content of any two homologous chromosomes was comparable and ranged between 44.5 and 48 (Table 2).

Using the Comparative Genome Viewer tool of NCBI, the two reference genomes were compared (Figure 1).

Chromosomes 1–4 of the two species were homologous, but with chromosome number 3 being inverted, i.e., the sequences of chromosome 3 were in two different directions. Chromosome 5 of *T. asperellum* is homologous to chromosome 6, chromosome 6 is homologous to chromosome 7, and chromosome 7 is homologous to chromosome 5 in *T. asperelloides* [Table 2; Figure 1]. We also observed clusters, mainly from the ends of the chromosomes, translocated to the ends or beginning of the same or different chromosomes, with the inversion highlighted by purple coloring (Figure 1).

### 3.2. Genes Coding for Lytic Enzymes and Secondary Metabolites

When the *T. asperellum* assembly genome was searched for chitinase using GDV, 22 hits were returned. The chitinase genes were present on all seven chromosomes (Table 3). Chromosome 7 had the most (five genes). The *T. asperelloides* chromosomes had 20 out of 22 *T. asperellum* hits, with homology between 93 and 97. *The asperelloides* lacked 9% of the chitinase genes present in *T. asperellum*. The genes are coding for proteins with mean sizes of 427, and a range of 309–947 amino acids. The chitinases belong to GH F18 according to the GenBank record for each gene. All GH F18 are chitinases according to the Glycoside Hydrolases family classification at https://www.cazy.org/Glycoside-Hydrolases.html (5 January 2026). The sequences of the chitinase genes from *T. asperellum* were downloaded, and alignments were attempted to determine if there are genes that exist as two copies. We found no similarities between the different genes, thus every gene is present as a single copy.

Searching the *T. asperellum* genome for cellulases, endoglucanase, exoglucanase, and 1,4 beta glucanase yielded 15 matches (Table 4). For each gene, a homologous gene was present in *T. asperelloides,* except for one case. The genes were distributed on all the chromosomes, except for chromosome number 3 (Table 4). The sizes of the putative proteins ranged from 246 to 739 amino acids.

Four and six genes were reported as endoglucanase and exoglucanase, respectively. Five genes were reported as 1,4-beta-D-glucanases. Among the latter, one gene was not present in *T. asperelloides*. The cellulases were from different Glycosyl Hydrolase (GH) families (Table 4). The DNA sequences of the cellulase genes were unrelated, and there were no duplicate genes.

The search for Xylanases in *T. asperellum* yielded two genes, XYN3 and XYN2. The two genes belong to the family of GH10 and GH11, respectively, and were located on chromosomes 6 and 3. Homologs to these two genes were present in *T. asperelloides* as well. Based on the Glycoside Hydrolases family, all the GH family numbers in Table 4 belong to cellulases and xylanases.

The search for secreted proteases in the genome of *T. asperellum* yielded 11 genes distributed on all the chromosomes (Table 5). The putative protein sizes ranged from 387 to 913 amino acids. Homologs to these genes were present in *T. asperelloides* with average identities of about 93–98% between any two corresponding genes. Alignment of the DNA sequences of these proteases showed no significant homology between them.

### 3.3. Genes Coding for NRPS, PkS, PkS-NRPS

The search in the genome of *T. asperellum* for genes coding for NRPS, PKS, and PKS-NRPS yielded a total of 54 matches for the three types of genes that were verified based on information in their GenBank records (Table 6). *T. asperelloides* lacked five (9%) of these genes involved in the biosynthesis of these specific secondary metabolites compared to *T. asperellum.* Within the NRPS and PKS-NRPS putative proteins in *T. asperellum*, six were implicated in the synthesis of siderophores based on the information in the GenBank record for those genes (Table 5). Siderophores have an affinity for turning insoluble iron (III) into a soluble complex available for the fungus and plants. Two of these genes were missing in *T. asperelloides* (Table 5). The DNA sequences of the *T. asperellum* genes, when aligned with the whole *T. asperellum* genome, showed no homology, meaning there were no duplicate genes.

The two species were also compared for genes involved in soil improvement and plant growth promotion. These genes were strongly conserved in the two species. The genome of *T. asperellum* had 26 genes coding for NADPH oxidase, laccases, urease, and acyl esterases. Homologs to these genes existed in one copy in the genome of *T. asperelloides* for 24 out of 26 genes (Table 7). The two missing genes were one for NADPH oxidase and one for laccases. The identity between homologous genes of the two species ranged from 91 to 98, and the description of the function of the enzymes coded by the genes has been given in the Material and Methods.

The summarized data for comparison of the genomes of *T. asperellum* and *T. asperelloides* for different genes involved in B&B activities are shown in Table 8. The two species have a comparable number of genes implicated in different activities, with a slight edge for *T. asperellum*. However, the total number of genes in the genome of *T. asperellum* was higher than that of *T. asperelloides* by about 1000 genes. However, as indicated in the methods, the data do not completely represent the differences in the number of genes involved in B&B activities in the two species, as the search for the genes in the *T. asperelloides* genome was not possible due to an unannotated genome.

### 3.4. Internal Transcribed Spacer (ITS) Region of rDNA

**Figure 2:**
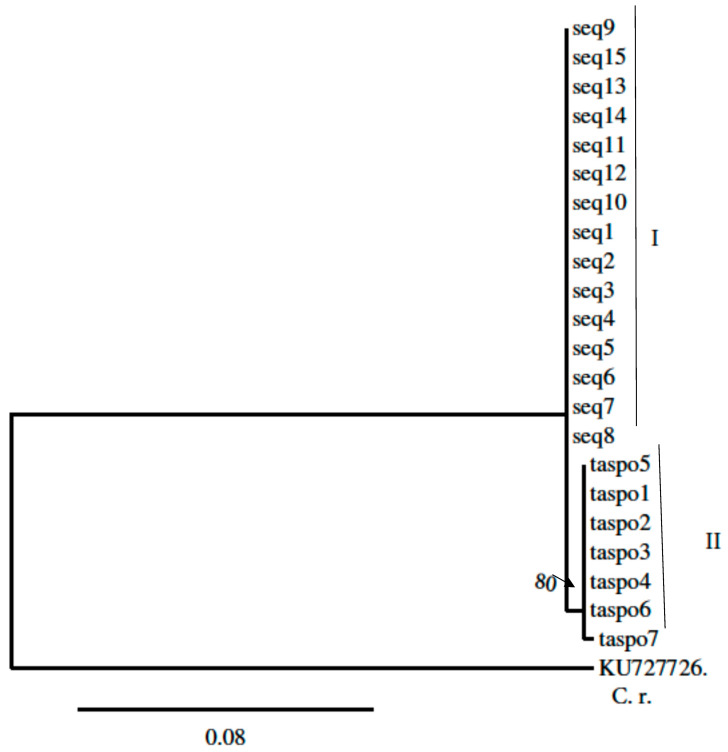
Maximum likelihood tree depicting the relationship of internal transcribed spacer (ITS) of the *T. asperellum* strain FT101 (Clade I) and the *T. asperelloides* strain X01119 (Clade II). The tree was rooted to the ITS of *Clonostachys rosa* (*C. r*.). = The number above the branch refers to the bootstrap value from 500 pseudo runs.

The search in the *T. asperellum* genome for the ITS region yielded 15 copies. All the copies were located on chromosome number seven. Eight of the copies had identical sequences. The rest of the copies (7) differed from the eight identical copies by one to four gaps, in different positions (Figure 2). Surprisingly, the genome of *T. asperelloides* only had seven copies of the ITS region, all located on chromosome number five. Four copies had identical sequences. The other three differed from the identical group of four by two gaps each in two different copies and by two nucleotides in another copy (Figure 2). The phylogenetic tree (Figure 2) also showed that *T. asperelloides* is derived relative to *T. asperellum* and thus *T. asperellum* is the more ancient species relative to *T. asperelloides*.

## 4. Discussion

Mycoparasitism has been found to be the original trait of *Trichoderma,* and it is the strategy for biocontrol when the prey is a plant pathogen [55]. Moreover, some *Trichoderma* species can infect or colonize the outer layers of roots or the rhizosphere region, which leads to positive responses in plants, such as growth promotion. This is the basis for the use of *Trichoderma* as a biofertilizer [55]. *Trichoderma* is likely the most studied of all biocontrol agents, with *Trichoderma atroviride* and *Trichoderma virens* being among the best mycoparasitic biocontrol agents used in agriculture [40] and among the most successful species in soil [10]. Like these two species, the two cryptic species *T. asperellum* and *T. asperelloides* are also dominant in soil and have been shown to be effective against various pathogens and in plant growth promotion based on previous research (Table 1). However, it is not clear which one of them has better biocontrol traits. In this study, the genomes of the two species were compared. The seven chromosomes were homologous with a 95% identity. We found chromosome number three for the two species to be homologous but inverted. This is due to the submission of the chromosome sequences in two different directions (note: GenBank staff, when consulted about this type of problem, confirmed that submitters can submit sequences in either direction, and there are no rules from GenBank on the subject, but only the submitters can change the sequence direction). The two genomes had sizes of 37.3 and 36.9 Mb for *T. asperellum* and *T. asperelloides*, respectively. They were comparable in size with two species, *T. virens* and *T. atroviride*, that are well-known for B&B activities [56]. However, the *T. asperellum* strain FT101 had 12,041 genes, whereas the *T. asperelloides* strain X01119 had 11,096 genes.

The two genomes were searched for genes coding for lytic enzymes implicated in mycoparasitism, such as chitinases, cellulases, xylanases, and proteases. These lytic enzymes have been reported as inducible during the interaction of *Trichoderma* with pathogenic fungi [57] and are the dynamic players in mycoparasitism or degradation of the host cell wall [58]. Results showed that *T. asperellum* had 22 genes coding for different chitinases. Homologs to 20 chitinase genes of *T. asperellum* were detected in *T*. *asperelloides.* All the genes belonged to the Glycosyl Hydrolase family 18 (GH18). Kubicek et al. [56] reported that *T. atroviride* and *T. virens* had 16 and 11 GH18 genes, respectively. The different chitinases in *T. asperellum* had no significant homology, indicating that each existed as one copy in the genome. Previously, chitinase inhibitory activity from the supernatant of cultures of *T. asperellum* against fungal pathogens was observed in in vivo and in vitro studies, implying direct roles of the chitinases in the biocontrol of *T. asperellum* [49].

In comparison to *T. asperellum*, *T. asperelloides* lacked only one out of 17 genes coding for different cellulases and xylanases; these two enzymes are used for breaking down the cellulose and hemicellulose, respectively, in cell walls of plants when living saprophytically. Cellulases are also used when *Trichoderma* infects and colonizes the outer layers of the roots and subsequently establishes communication with the plant, leading to induced systemic disease resistance [9]. Specifically, *T. asperellum* has been shown to induce systemic resistance (ISR) against *Pseudomonas syringae* pv. *Lachrymans*, the causative agent of angular leaf spot of cucumber. The ISR was associated with an arrest in the proliferation of the bacteria, increased phenolic secondary metabolites, and induced expression of two defense genes in the cucumber seedlings [19].

Pathogens belonging to Oomycetes, like *Phytophthora* and *Pythium,* have cellulose in their cell walls [59]. Cellulase activity was produced by *Trichoderma* spp. During the mycoparasitism of *Phytophthora capsici*, the causative agent of root rot of black pepper [60].

We limited the comparison of proteases to secreted proteases that are used in mycoparasitism. The two species had the same number (11 in each) of secreted protease genes of different types and sizes. Secreted proteases have been implicated in the degradation of the cell wall of nematodes at different growth stages [61].

Nonribosomal peptide synthetases (NRPSs), polyketide synthases (PKSs), and fused PKS-NRPS synthases are multi-enzymatic, multi-domain mega-synthases involved in the biosynthesis of non-ribosomal peptides and polyketides, e.g., peptaibols and siderophores. These secondary metabolites exhibit a remarkable array of biological activity, and many of them are clinically valuable anti-microbial, anti-fungal, anti-parasitic, anti-tumor, and immunosuppressive agents [62]. The total number of NRPS, PKS, and PKS-NRPS genes was 54 and 49 in *T. asperellum* and *T. asperelloides*, respectively. Five genes present in *T. asperellum* were missing in *T. asperelloides*. *T. virens* and *T. atroviride* had 50 and 35 of these genes, respectively [56]. The putative proteins for six genes (Table 6) were implicated for the synthesis of iron (III) chelating proteins, siderophores, according to the GenBank record, associated with the genes. Thus, the success of *T. asperellum* and *T. asperelloides* in soil could involve limiting the availability of iron for pathogens and competitor fungi. These siderophores would also render iron available for plant growth. Surprisingly, two of the five missing secondary metabolite coding genes (40%) in *T. asperelloides* were among the six genes involved in siderophore synthesis. More efficacy experiments under different iron conditions are needed to prove whether the two missing genes implicated in the siderophore synthesis could cause any difference in the B&B activities of *T. asperelloides* compared to *T. asperellum*. *T. asperellum* strain Q1 was shown to be a siderophore producer that had high affinity for iron (III) and promoted growth of *Arabidopsis thaliana* in an iron-deficient or insoluble iron-containing (Fe_2_O_3_) medium [63]. The biocontrol and biofertilizer effects of siderophores have been studied in other species of *Trichoderma*. *T. harzianum* has been shown to produce a siderophore that inhibited the growth of *Rhizoctonia solani* [64]. In *T. virens,* on the other hand, the fungus produced siderophores, which inhibited infection of *Fusarium oxysporum* and promoted growth in banana plants [65].

Like the genes involved in biocontrol, the genes involved in soil and plant health were highly conserved between the two species, *T. asperellum* and *T. asperelloides*. The two species shared 24 out of 26 genes (92%). Many mechanisms for improved plant growth by *Trichoderma* have been proposed, including production of phytohormones, the solubilization of sparingly soluble minerals, the induction of systemic resistance in the host plant, a reduction in pollutant toxicity, organic or heavy metal, and the regulation of rhizospheric microflora [66,67,68,69,70]. Therefore, it appears there are many genes involved in the process without dominant genes involved, such as in mycoparasitism and antibiosis.

As the results showed, there are many different chitinases, cellulases, proteases, and other enzymes involved in the biosynthesis of secondary metabolites and those involved in soil and plant health. Even though the genes encoding them were located on different chromosomes, a study showed that many of them could be transcribed together. When *Trichoderma asperellum* interacted with the plant (*Populus davidiana* × *P. alba var. pyramidalis),* in one of the four transcriptomes, 12 chitinases and five glucanase genes were highly expressed, indicating that mycoparasitism genes could be expressed upon exposure to plants and before interacting with the pathogens [71]. In another study, the treatment of tomato plants with *T. asperelloides* under drought conditions caused increased expression of secondary metabolite genes, implying that secondary metabolites are not only involved in biocontrol against pathogens, but can help plants under abiotic stress conditions like water deficit [72]. Transcriptome studies have also revealed that biocontrol strategies differ in different *Trichoderma* species. Upon confrontations with *Rhizoctonia solani*, *T. atroviride* had differential expressions of genes involved in the production of secondary metabolites, GH16 ß-glucanases, various proteases, and small secreted cysteine-rich proteins. *T. virens*, on the other hand, had a higher expression of genes coding for the synthesis of gliotoxin, its precursors, and glutathione, which is necessary for the synthesis of gliotoxin. In contrast, *T. reesei* increased the expression of genes encoding cellulases and hemicellulases, and of the genes involved in solute transport [73].

We also analyzed the number of ITS regions of the rDNA cluster. *T. asperellum* has 15 ITS regions, all on chromosome seven, compared to seven regions in *T. asperelloides* on chromosome five. The number of ITS region copies for the *T. atroviride* assembly genome (ASM2064779v1) was 10, which is lower than that of *T. asperellum* but greater than for *T. asperelloides*. The genomes of *T. reesei* (GCF_001167675.1), a cellulolytic industrial species, and *T. longibrachiatum* (ASM2625927v1) (not known for biocontrol purposes) had one and four copies of the ITS locus, respectively. These numbers are substantially fewer than the copy number for the known biocontrol species. It is noteworthy to mention that the whole-genome sequences above for *T. longibrachiatum* and *T. reesei* were not assembled to the chromosome level, and the ITS copy numbers may not be reliable. Further comparisons between different strains for these two species are needed to show if the number of ITS regions observed in this study is valid for all the strains. At this stage, there are not enough whole-genome sequences from both species, especially *T. asperelloides*, to make such a comparison. The large-scale comparisons between species in different sections of the genus may reveal phylogenetic and biological significance of the ITS/rDNA copy number.

This study also showed that not all the ITS copies in one species are identical in DNA sequence, even though all the ITS sequences deposited in GenBank for *Trichoderma* fungi report the ITS sequence for any strain as one sequence, identical for all the copies in the genome. For those involved in phylogenetic studies of closely related species or cryptic species, we suggest that they look at the ITS in the whole genomes as well.

In summary, the genomic comparison of the two cryptic species *T. asperellum* and *T. asperelloides* revealed that *T. asperellum* had a greater number of total genes and a slightly higher number of genes encoding enzymes involved in mycoparasitism, the synthesis of secondary metabolites, and soil and plant health, along with a higher ITS/rDNA copy number than that of *T. asperelloides*. However, the data collected in this study may not reflect the full comparison of key genes in B&B since the number of genes present in *T. asperelloides*—but not in *T. asperellum*—was not evaluated due to a lack of annotation of the *T. asperelloides* genome. The slight differences in genes coding for B&B enzymes between these two species may be resolved by future functional studies.

To connect the number of strains reported in GenBank with the data in this study, we searched GenBank (accessed on 15 April 2026) for *Trichoderma asperellum* and *T. asperelloides* separately, and the number of matches for the two species was 24,233 and 1144, respectively. Even though these numbers were not part of a statistically designed survey study, the huge difference (21-fold) may point to *T. asperellum* being more frequently isolated from soil and other environmental niches than *T. asperelloides.* Whether the genomic differences observed in this study have an effect on the dominance of one species over the other in their habitats (especially soil) remains to be answered in future studies on transcriptomes, proteomes, ribosome biogenesis, enzyme activity, or biological efficacy studies of the two species.

Are all strains of *T. asperellum* or *T. asperelloides* equally good for biocontrol? According to Harman [9], the answer is no, not all strains of a species are good for biocontrol. The phylogenetic analyses of populations of *T. asperellum* strains [10,11] clearly reveal high diversity within this species, as shown by the many clades and subclades in the tree. It is possible that those clades/subclades will exhibit biocontrol specificity with certain plants and diseases because of adaptation and co-evolution. Phylogenetically, *T. asperelloides* strains are less diverse than *T. asperellum* [10], which could be due to the fact that *T. asperellum* is the more ancient of the two species.

We also emphasize that the genomic comparison presented in this study was based on sequences of one strain for each species. However, the two sequences were assembled to the chromosome level and are considered reference sequences for the two species in GenBank. There are hundreds of sequences of whole genomes for *Trichoderma* in GenBank; however, sequences assembled to the chromosome level are rare and do not exceed 20. Among the chromosome-level sequences, other unresolved concerns remain, for example, chromosome numbering and the direction of the sequences. To correct these issues, we suggest that rules for chromosome sequence direction and chromosome numbering be established based on specific gene(s) in each chromosome to avoid random numbering or numbering based on the sizes of chromosomes.

The homology between the chromosome sequences of the two strains of *T. asperellum* that were assembled to the chromosome level was evaluated using BLAST as described in Section 2.1 to determine intraspecies variability in *T. asperellum*. Identities between the five homologous chromosomes of *T. asperellum* FT101 (used in this study) and *T. asperellum* DQ-1 assembly number (ASM17945596v1) were 98%. Based on previous studies, the diversity within the population of *T. asperelloides* strains was lower than the diversity within the population of *T. asperellum* strains. Therefore, chromosome sequence identity between *T. asperelloides* strains could be as high as the identity within *T. asperellum* strains (98%). Consequently, we feel that the results of this study on the genomic comparison of two strains can be extended to all the strains of the two species. However, further comparisons of whole genomes of more strains will be essential to prove whether the differences in the number of genes obtained in this study will be valid for all the strains of the two species. Such comparisons will be possible when more whole-genome sequences assembled to the chromosome level are available in GenBank.

## 5. Conclusions

This is the first comparative genomic study to focus exclusively on genes involved in B&B activities of *Trichoderma* species. The study revealed that within *Trichoderma,* cryptic species may differ genomically in general and in B&B-involved genes, as evidenced by the greater number of total genes and slightly higher genes involved in biocontrol in *T. asperellum* than were present in *T. asperelloides*. Further transcriptomic, proteomic, ribosome biogenesis, enzyme activity, or biological efficacy studies can elucidate the significance of the differences. The methods used in this comparative study are reproducible and do not require any specific software other than that freely available in NCBI. Specifically, the methods can be used to differentiate other cryptic species in the *Trichoderma,* particularly in the Harzianum Clade, the position of one of the most important biocontrol species, *T. afroharzianum* T 22 [74]. This is also the first study to reveal differences in ITS/rDNA copy numbers in the genomes of the two cryptic species, though the biological and phylogenetic significance of the finding needs further study. The study had two limitations. First, the search for B&B genes in *T. asperelloides* was not possible due to a lack of annotation of the genome. Thus, the study only shows comparisons between B&B genes present in *T. asperellum* and whether the homologs of each gene were present or not in *T. asperelloides*. Second, GenBank lacks high-quality genomes (assembled to the chromosome level and annotated) of *Trichoderma* species for more strains of these two species to show if the comparison applies to all the strains of the two species. Therefore, we conclude that there is an urgent need for more *Trichoderma* whole-genome sequences assembled to the chromosome level and annotated in GenBank.

## Figures and Tables

**Table 1:** Biocontrol studies on *T. asperellum* and *T. asperelloides*.

Function	Plants	Reference
***T. asperellum***		
- Induction of systemic resistance against angular leaf spot caused by (*Pseudomonas syringae* pv. *lachrymans*)	Cucumber	[19]
- Control of root rot disease caused by *Pythium myriotylum*	Cocoyam	[20]
- Controlling vascular dieback streak disease caused by *Ceratobasidium theobromae*	*Theobroma cacao*	[21]
- Controlling wilt caused by *Fusarium oxysporum*	Tomato	[22]
- Reducing white rot caused by *Sclerotium cepivorum*	Onion	[23]
- Inhibitory activity (in vitro) against fungal pathogens	NA	[24]
- Promoted growth of roots	Watermelon, tomato, eggplant, chili	“
- Increased seed germination	Rice, cucumber, tomato, melon, pak choi	“
- Plant growth promoter, suppressor of wilt caused by *F. oxysporum*	Tomato	[25]
- Plant growth promotion, antifungal against wilt disease	Maize	[26]
- Controlling wilt caused by *F. oxysporum*	Cavendish banana	[27]
- Effective in reducing rhizoctonia root rot and clubroot severity in greenhouse	Radish	[28]
- Plant growth promotion	Apple	[29]
- *Fusarium* wilt control	Watermelon	[30]
- Controlling collar rot caused by *Agroathelia rolfsii*	Tomato	[31]
- Effective against anthracnose caused by *Colletotrichum gleosporioides*	Chili pepper	[32]
- Control of black spot disease caused by *Alternaria alternata*	Pear	[33]
***T. asperelloides***		
- Control of white mold disease caused by *Sclerotinia sclerotiorum*	Soybean	[34]
- Biocontrol and promotion of growth	*Arabidopsis thaliana*	[35]
- Control of gummy stem blight caused by *Stagonosporopsis cucurbitacearum*	Muskmelon	[36]
- In vitro suppression of five emerging fungal pathogens by volatile metabolites	N/A	[37]
- Effective in controlling Grapevine dieback of the trunk caused by *Botryosphaeria*	Grapevine	[38]
- Effective in reducing *Rhizoctonia* root rot and clubroot severity in greenhouse	Radish	[28]
- Induced water stress tolerance	Tomato	[39]
- Effective against anthracnose caused by *C. gleosporioides*	Chili pepper	[32]

**Table 2:** General comparison of *Trichoderma asperellum* and *Trichoderma asperelloides* genome.

*T. asperellum*				*T. asperelloides*			Comparison
Chro. #	Chro. Accession #	Size (nt)	GC%	Chro. Accession #	Size (nt)	GC%	Identity%
1	CP084943.1	7,308,209	48	CM125459.1	7,077,426	48	95.43
2	CP084944.1	7,001,256	48.5	CM125460.1	7,036,127	48	94.94
3	CP084945.1	5,512,738	48	CM125461.1	5,492,770	48	95.00
4	CP084946.1	5,435,626	46.5	CM125462.1	5,294,182	47	94.98
5	CP084947.1 ^a^	4,134,283	46	CM125463.1	4,073,944	46	95.08
6	CP084948.1	4,132,110	46.5	CM125464.1 ^a^	4,017,442	47	95.22
7	CP084949.1	4,021,158	44.5	CM125465.1	3,875,434	46.5	94.64
MT ^b^	CP084950.1	30,285	28				
Total size (mB)		37.3			36.9		
Total genes		12,041			11,096		

^a^ chromosomes with the same color in the two species are homologous. ^b^ MT refers to mitochondria.

**Table 3:** Chitinase coding genes present in *Trichoderma asperellum* and their homologs in *Trichoderma asperelloides*.

*T. asperellum*				*T. asperelloides*	Comparison	
Gene Code	Gene Type	Chro ^d^. #	Length (aa)	Chro. #	Identity	Direction
CHIT46	GH 18	4	424	4	97%	P/P ^b^
CHIT33	GH 18	1	322	1	96%	P/P
CHI3_2	GH 18	7	410	5	97%	P/P
CHI3_1	GH18	2	309	2	96%	P/P
CHI2_3 ^a^	GH 18	4	404	4	94%	P/P
				4	93%	P/P
CHI2_4 ^a^	GH 18	4	326	4	94%	P/P
				4	93%	P/P
CHI2_1	GH 18	1	416	1	95%	P/P
CHIB1	GH 18	2	397	2	97%	P/P
CHIT37	GH 18	5	344	6	96%	P/P
CHI2_2	GH 18	3	408	3	97%	P/P
TrAFT101_008694	GH 18	5	337	6	92%	P/P
TrAFT101_003388	GH 18	2	538	2	97%	P/P
TrAFT101_004622	GH 18	3	397	3	94%	P/M
TrAFT101_010304	GH 18	6	947	7	94%	P/P
TrAFT101_010810	GH 18	7	392	5 (- ^c^)		
TrAFT101_010889	GH 18	7	362	5	92%	P/P
TrAFT101_011632	GH 18	7	397	5	95%	P/P
TrAFT101_005436	GH 18	3	395	3	95%	P/M
TrAFT101_005299	GH 18	3	357	3	98%	P/M
TrAFT101_003370	GH 18	2	371	2	94%	P/P
TrAFT101_011669	GH 18	7	688	5	94%	P/P
TrAFT101_009462	GH 18	5	457	6 (-)		

^a^ BLAST showed two regions of homology; ^b^ P/P, genes in the two species are in the same direction, and P/M, genes in the two species are in opposite directions; ^c^ -, no homolog detected in *T. asperelloides*; ^d^ chrom., chromosome number.

**Table 4:** Cellulase coding genes in *Trichoderma asperellum* and their homologs in *Trichoderma asperelloides*.

*T. asperellum*				*T. asperelloides*	Comparison	
Gene	Gene Family	Chro. #	Length (aa)	Chro. #	Identity	Direction
**Endoglucanase**						
EGL2_1	GH5 ^a^	4	425	4	98%	P/P ^b^
EGL1	GH7	6	467	7	92%	P/P
EGL2_2	GH5	4	418	4	96%	P/P
EGL5	GH45	2	248	2	95%	P/P
**Exoglucanase**						
CBH2	GH6	4	470	4	96%	P/P
CBH1	GH7	6	507	7	97%	P/P
TrAF101-010989	GH5	7	442	5	93%	P/P
TrAF101-008251	GH12	5	327	6	94%	P/P
TrAF101-010504	GH12	6	234	7	90%	P/P
TrAF101-009491	GH12	6	246	7	95%	P/P
**1,4-beta-D-glucanase**						
TrAFT101_001003	GH16	1	739	1	93%	P/P
TrAFT101_007062	GH16	4	751	4	96%	P/P
TrAFT101_003630	GH16	2	335	2	94%	P/P
TrAFT101_010800	GH16	7	337	5 (- ^c^)		
TrAFT101_008744	GH16	5	367	6	93%	P/P
**Xylanase**						
XYN3	GH10	6	346	7	93%	P/P
XYN2	GH11	3	223	3	94%	P/M

^a^ GH refers to Glycosyl Hydrolase; ^b^ P/P, genes in the two species are in the same direction, and P/M, genes in the two species are in opposite direction; and ^c^ -, no homolog gene detected in *T. asperelloides*.

**Table 5:** Secreted protease coding genes in *Trichoderma asperellum* and their homologs in *Trichoderma asperelloides*.

*T. asperellum*				*T. asperelloides*	Comparison	
Gene Code	Gene Type	Chro. #	Length (aa)	Chro. #	Identity	Direction
KEX1	Serine carboxypeptidase	4	630	4	96%	P/P ^a^
TrAFT101_004526	Peptidase S8	2	882	2	98%	P/P
TrAFT101_000377	Peptidase S8	1	924	1	94%	P/P
TrAFT101_003852	Peptidase S8	2	913	2	94%	P/P
TrAFT101_001752	Serine protease	1	255	1	96%	P/P
TrAFT101_011675	Pepsin-retropepsin-like	7	355	5	96%	P/P
TrAFT101_000300	Pepsin-retropepsin-like	1	387	1	96%	P/P
TrAFT101_002525	Protease-like-domain	2	824	2	95%	P/P
TrAFT101_009555	Zinc-peptidase-like	6	760	7	93%	P/P
TrAFT101_004497	Zinc-peptidase-like	2	492	2	93%	P/P
TrAFT101_005887	Peptidase S8	3	444	3	95%	P/M

^a^ P/P, genes in the two species are in the same direction, and P/M, genes in the two species are in opposite directions.

**Table 6:** Genes coding for PKS, NRPS, and Fused PKS-NRPS in *Trichoderma asperellum* and their homologs in *Trichoderma asperelloides*.

*T. asperellum*				*T. asperelloides*	Comparison	
Gene Code	Gene	Chro. #	Length (aa)	Chro. #	Identity	Direction
TrAFT101_004419	NRPS	2	1285	2	96%	P/P ^a^
TrAFT101_008186	NRPS	5	1020	6	95%	P/P
TrAFT101_010145 ^c^	NRPS	6	1812	7	96%	P/P
TrAFT101_004574 ^c^	NRPS	2	3267	2	96%	P/P
TrAFT101_011349	NRPS	7	527	5	92%	P/P
TrAFT101_009885	NRPS	6	7417	7	96%	P/P
TrAFT101_004573	NRPS	2	2912	2	95%	P/P
TrAFT101_009644	NRPS	6	8245	7	94%	P/P
TrAFT101_008129	NRPS	4	927	4	94%	P/P
TrAFT101_011936	NRPS	7	5894	5	96%	P/P
TrAFT101_004614	NRPS	3	2810	3 (- ^b^)		
TrAFT101_008346	NRPS	5	2148	7	94%	P/P
TrAFT101_000035 ^c^	NRPS	1	5458	1 (-)		
TrAFT101_008259	NRPS	5	4551	6	94%	P/P
TrAFT101_006466	NRPS	4	1687	4	95%	P/P
TrAFT101_006548	NRPS	4	4870	4	95%	P/P
TrAFT101_003860	NRPS	2	3143	2	93%	P/P
TrAFT101_111350	NRPS	7	552	5	92%	P/P
TrAFT101_005171	NRPS	3	1591	3	98%	P/M
TrAFT101_008191	NRPS	5	189	6	98%	P/P
TrAFT101_002349	NRPS	2	380	2	95%	P/P
TrAFT101_010456	NRPS	6	599	7	93%	P/P
TrAFT101_011231	NRPS	7	1223	5	94%	P/P
TrAFT101_011933	NRPS	7	581	5	95%	P/P
TrAFT101_010144	NRPS	6	574	7	96%	P/P
TrAFT101_002348	NRPS	2	524	2	95%	P/P
TrAFT101_002608	NRPS	2	1169	2	96%	P/P
TrAFT101_000168	NRPS	1	1159	1	95%	P/P
TrAFT101_000108	NRPS	1	1621	1	94%	P/P
TrAFT101_002088	NRPS	1	848	1	96%	P/P
TrAFT101_002272	NRPS	1	1010	1	94%	P/P
TrAFT101_011006 ^c^	NRPS	7	1160	5 (-)		
TrAFT101_006496	NRPS	4	1055	4	97%	P/P
TrAFT101_008343	NRPS	5	1051	6	94%	P/P
TrAFT101_000207	Iterative PKS	1	3133	1	93%	P/P
TrAFT101_010457	Iterative PKS	6	2442	7	92%	P/P
TrAFT101_000373	Iterative PKS	1	2317	1	94%	P/P
TrAFT101_011200	Iterative PKS	7	2341	5	95%	P/P
TrAFT101_009627	Iterative PKS	6	1268	7	94%	P/P
TrAFT101_006398	Iterative PKS	3	1799	3	94%	P/M
TrAFT101_012069	Iterative PKS	7	2437	5	96%	P/P
TrAFT101_008352	Iterative PKS	5	2489	6	96%	P/P
TrAFT101_010217	Iterative PKS	6	2364	7	94%	P/P
TrAFT101_008421	Iterative PKS	5	2530	6	96%	P/P
PKS1	PKS	6	2154	7 (-)		
TrAFT101_008181	PKS	5	1786	6	95%	P/P
FUB1_2	PKS	2	1112	2	97%	P/P
TrAFT101_000134	PKS	1	407	1	88%	P/P
TrAFT101_009882 ^c^	PKS-NRPS	6	10,410	7	95%	P/P
TrAFT101_006283 ^c^	PKS-NRPS	3	12,227	3	96%	P/M
TrAFT101_012067	PKS-NRPS	6	2306	7 (-)		
TrAFT101_000054	PKS-NRPS	1	4070	1	99%	P/M
TrAFT101_011929	PKS-NRPS	7	768	5	93%	P/P
TrAFT101_011931	PKS-NRPS	7	1627	5	95%	P/P

^a^ P/P, genes in the two species are in the same direction, and P/M, genes in the two species are in opposite directions. ^b^ -, no homolog gene detected in *T. asperelloides*. ^c^ putative protein involved in the synthesis of siderophores.

**Table 7:** Genes involved in biofertilizer function in *Trichoderma asperellum* and their homologs in *Trichoderma asperelloides*.

	*T. asperellum*		*T. asperelloides*	Comparison	
Gene Code	Chro. #	Length (aa)	Chro. #	Identity	Direction
**NADPH oxidase**					
TrAFT101_002743	2	570	2	96%	P/P ^a^
TrAFT101_005835	3	557	3	97%	P/M
TrAFT101_011647	7	506	5	96%	P/P
TrAFT101_006437	4	539	4	98%	P/P
TrAFT101_006420	4	352	4 (- ^b^)		
TrAFT101_003112	2	737	2	96%	P/P
TrAFT101_007182	4	695	4	93%	P/P
TrAFT101_009683	6	634	7	91%	P/P
TrAFT101_009906	6	621	7	96%	P/P
TrAFT101_009939	6	831	7	97%	P/P
TrAFT101_002964	2	590	2	96%	P/P
TrAFT101_005587	3	790	3	96%	P/M
TrAFT101_011062	7	644	5	95%	P/P
TrAFT101_009386	5	731	6	96%	P/P
TrAFT101_007280	4	554	4	93%	P/P
TrAFT101_004504	2	637	2	92%	P/P
**Laccases**					
TrAFT101_006462	4	566	4	97%	P/P
TrAFT101_011088	7	603	5	95%	P/P
TrAFT101_09790	6	590	7	95%	P/P
MLAC1	6	614	7 (-)		
**Urease**					
URE1	3	835	3	93%	P/M
TrAFT101_008952	5	330	6	95%	P/P
TrAFT101_001380	1	560	1	96%	P/P
**Acyl esterases**					
TrAFT101_007109	4	407	4	95%	P/P
TrAFT101_000427	1	1055	1	97%	P/P
TrAFT101_008568	5	339	6	94%	P/P

^a^ P/P, genes in the two species are in the same direction, and P/M, genes in the two species are in opposite directions. ^b^ -, no homolog detected in *T. asperelloides*.

**Table 8:** Comparison of the number of genes for different biocontrol and biofertilizer activities in two cryptic species of *Trichoderma*.

Activity	*T. asperellum*	*T. asperelloides*
Mycoparasitism ^a^	48	45
Antibiosis ^b^	54	49
Soil and plant health ^c^	26	24
Total ^d^	12,041	11,096

^a^ the mycoparasitism genes included: chitinases, cellulases, xylanases, and secreted proteases from Table 3, Table 4 and Table 5. ^b^ the antibiosis genes included: NRPS, PKS, and PKS-NRPS in Table 6. ^c^ the soil and plant health genes included: NADPH oxidase, laccases, urease, acyl esterases in Table 7. ^d^ the total genes obtained from Table 2.

## Data Availability

Data is contained within the article and Supplementary Materials.

## Supplementary Materials

The following supporting information can be downloaded at https://www.mdpi.com/article/10.3390/jof12060418/s1. Figure S1. Comparison of genomes of *T. asaperellum* and *T. asperelloides* using Comparative Genome Viewer tool of NCBI. Figure S2. *T. asperellume* genome opened in Genome Data Viewer of NCBI then search for “chitinase” and click on any hit e.g., CHI2_4 and the gene appears in green. Figure S3. BLAST with a chitinase encoding gene in *T. asperellum* on chromosome 4 as Query Sequence against entire chromosome 4 in *T. asperelloides* as Subject sequence. Figure S4. The first step in search for ITS regions in *T. asperellum* genome is selection of BLAST the reference genome. Figure S5. Search for ITS regions in genome of *T. asperellum*.
