# Supplementary material for: Trichoderma asperellum and T. asperelloides: Comparative Genomic Study for Genes Implicated in Biocontrol and Biofertilizer Activities
Source: J Fungi (Basel). 2026 Jun 9;12(6):418. doi: 10.3390/jof12060418 (PMC13301806; doi:10.3390/jof12060418)
Supplement: Supplementary file 1 [file jof-12-00418-s001.zip › figure S2.pdf]

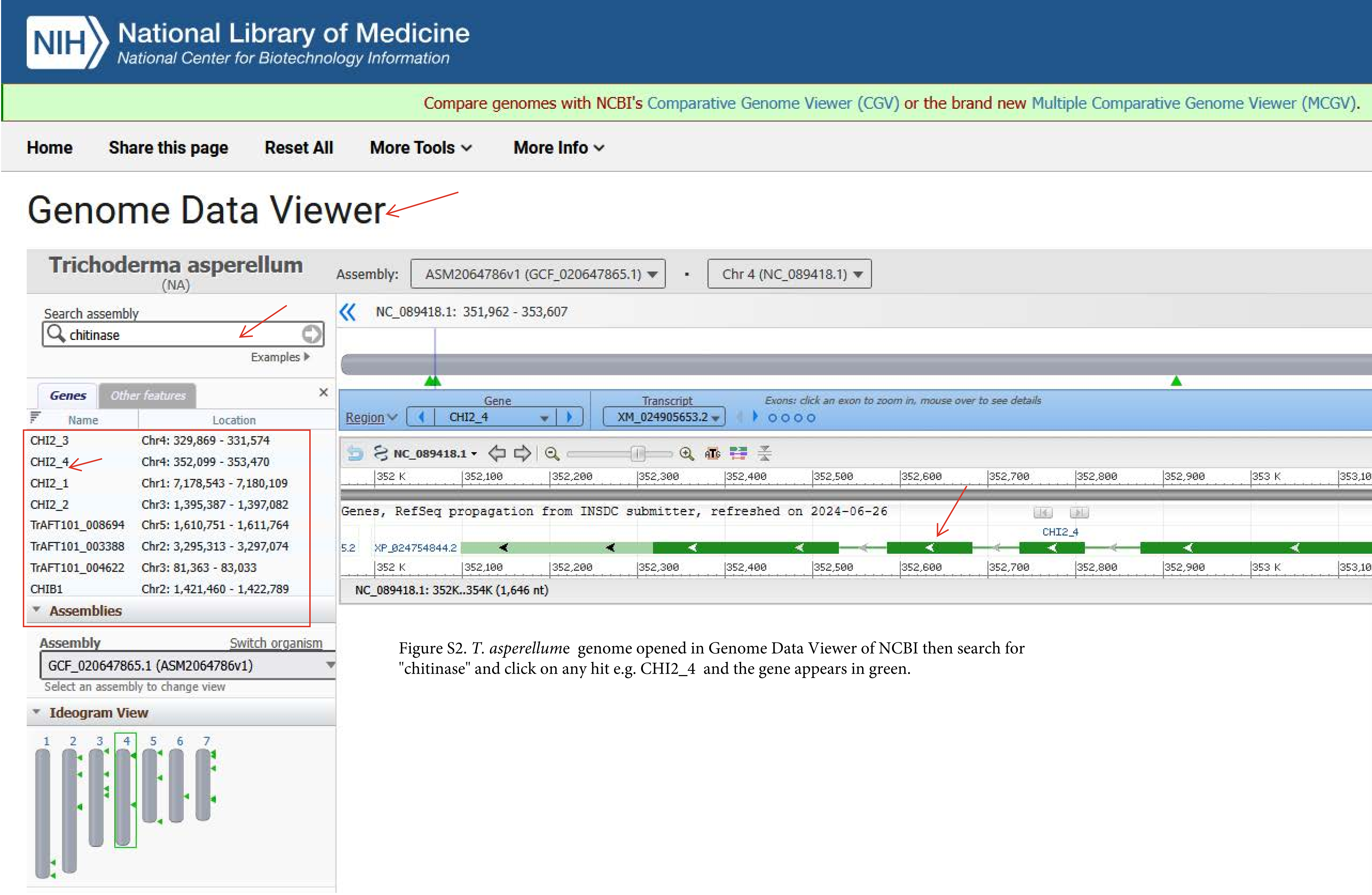

Figure S2. *T. asperellume* genome opened in Genome Data Viewer of NCBI then search for "chitinase" and click on any hit e.g. CHI2_4 and the gene appears in green.
