# Supplementary material for: Trichoderma asperellum and T. asperelloides: Comparative Genomic Study for Genes Implicated in Biocontrol and Biofertilizer Activities
Source: J Fungi (Basel). 2026 Jun 9;12(6):418. doi: 10.3390/jof12060418 (PMC13301806; doi:10.3390/jof12060418)
Supplement: Supplementary file 1 [file jof-12-00418-s001.zip › Figure S5.pdf]

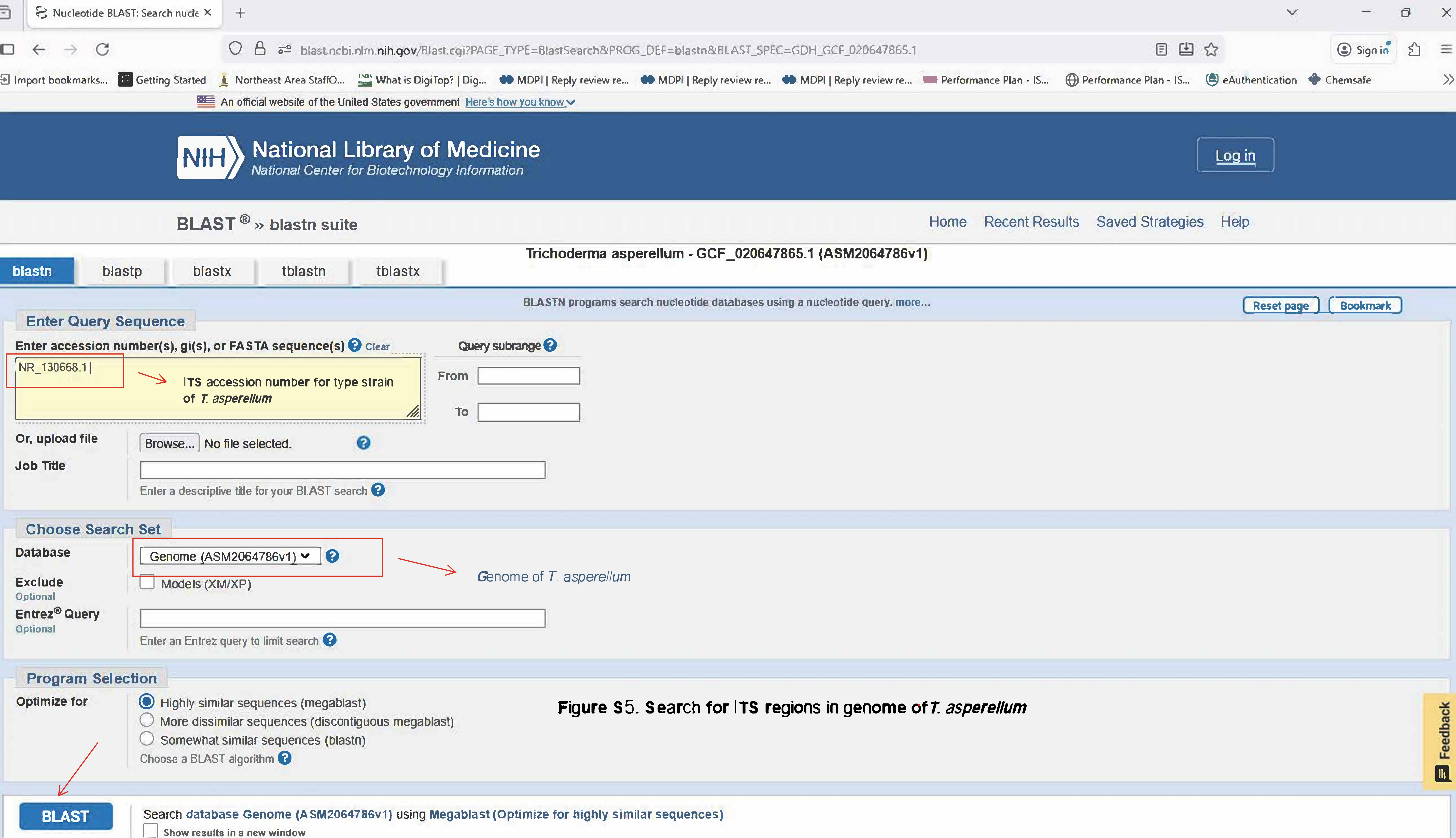

Figure S5. Search for ITS regions in genome of *T. asperellum*
